# Zeros of Planar Ising Models via Flat SU(2) Connections

**DOI:** 10.1007/s00220-025-05516-x

**Published:** 2025-12-06

**Authors:** Marcin Lis

**Affiliations:** Technische Universität Wien, Vienna, Austria

## Abstract

Livine and Bonzom recently proposed a geometric formula for a certain set of complex zeros of the partition function of the Ising model defined on planar graphs Livine and Bonzom (Phys Rev D 111(4):046003, 2025). Remarkably, the zeros depend locally on the geometry of an immersion of the graph in three dimensional Euclidean space (different immersions give rise to different zeros). When restricted to the flat case, the weights become the critical weights on circle patterns Lis (Commun Math Phys 370(2):507–530, 2019).We rigorously prove the formula by geometrically constructing a null eigenvector of the Kac–Ward matrix whose determinant is the squared partition function. The main ingredient of the proof is the realisation that the associated Kac–Ward transition matrix gives rise to an $\text {SU}(2)$ connection on the graph, creating a direct link with rotations in three dimensions. The existence of a null eigenvector turns out to be equivalent to this connection being flat.

## Introduction and Main Result

Let $\mathcal G=(\mathcal V, \mathcal E)$ be a finite graph. Given real coupling constants $\mathcal J: \mathcal E\rightarrow \mathbb R$ and external fields $h:\mathcal V\rightarrow \mathbb R$, the Ising model on $\mathcal G$ is a probability measure on the space of spin configurations $\{-1,+1\}^\mathcal {V}$ given by

$$\begin{aligned} \mathbb {P}(\sigma ) = \frac{1}{\mathcal { Z} } \exp \Big ( \sum _{\{v,v'\}\in \mathcal { E}} \mathcal { J}_{\{v,v'\}} \sigma _v \sigma _{v'} + \sum _{v\in \mathcal { V}} h_v \sigma _v \Big ), \end{aligned}$$

where $\mathcal Z=\mathcal Z_\mathcal {G}(\mathcal J,h)$ is the normalising constant, called the *partition function*. We will write $\mathcal Z_\mathcal {G}(\mathcal J)=\mathcal Z_\mathcal {G}(\mathcal J,0)$ for the case with no external field, which is the main focus of this work. Various thermodynamic quantities of the model are expressed through the derivatives of the *free energy* $\mathcal F:=\ln \mathcal Z$ with respect to the given parameters. As long as the parameters are real and the graph finite, the partition function is a positive analytic function, and hence all the derived quantities of interest are analytic as well. Therefore, to detect a phase transition one has to take limits of increasing graphs, which leads to the zeros of the partition function, now seen as a function of complex parameters, approaching the real line. In turn this causes the lack of analyticity of the free energy and a phase transition occurs. The study of the set of zeros of the partition function is therefore of importance for the understanding of the phase transition of the model. As such it has a rich and successful history, which goes back to the seminal work of Lee and Yang [24] who showed that all zeros of $\mathcal Z$, when viewed as a function of the external field *h* only, are purely imaginary. This was later extended to more general spin systems [25, 32, 36]. These results hold irrespectively of the underlying finite graph (in particular no assumption about planarity is necessary).

On the other hand the zeros in the complex coupling constants $\mathcal J$, also called *Fisher zeros* after [15], are less understood with no general theory available (see [30] and the discussion therein). In their recent work, Livine and Bonzom [31], using partially nonrigorous arguments (that among others include a rigorous mapping between the Ising model and spin networks [4], and a saddle point analysis of the resulting series), proposed a parametrisation of a certain class of Fisher zeros for Ising models defined on planar graphs. Surprisingly the parametrisation concerns the geometry of embeddings (or as it turns out immersions) of the graph into the three dimensional Euclidean space. The planar Ising model is more than a hundred years old [20], and it has been known to be exactly solvable since the groundbreaking work of Onsager [33] in the 1940’s. Moreover its relation to two dimensional discrete geometry has been extensively studied in recent years in connection to conformal invariance at criticality [7, 8, 10, 23, 37]. However, to the best of our knowledge, the appearance of three dimensional discrete geometry is very much unexpected and no such formula had been proposed before. We note that when the immersion is locally flat (meaning that a part of it is contained in a plane), then the weights on that part become the critical Ising weights on circle pattern tilings of the plane that were introduced by the author in [26]. Here criticality means that after the introduction of an additional parameter – the inverse temperature $\beta >0$, that scales all the coupling constants simultaneously – the model has exponential decay of correlations for $\beta <1$ and positive magnetisation for $\beta >1$.

**Fig. 1:**
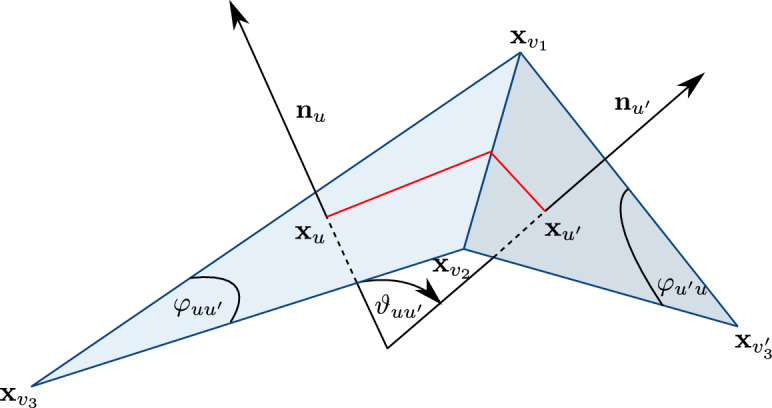
Two adjacent faces of an immersion $\textbf{x}$ of *G*. The triangles $\textbf{T}^\textbf{x}_u$ and $\textbf{T}^\textbf{x}_{u'}$ are represented by the light blue shading. The red piecewise linear segment represents the edge $\{u,u'\}$ in $G^*$

In this note we give a rigorous proof of the formula of Livine and Bonzom. To state the result, we need to introduce the setup. To this end, let $G=(V,E)$ be now a finite connected *planar* graph and let $G^*=(U,E^*)$ be its planar dual. We assume that *G* is a triangulation (and hence $G^*$ is trivalent). The results generalise to arbitrary planar graphs in a way described in Remark 1. We will identify each face *u* of *G* (vertex of $G^*$) with the triple of vertices $\{v_1,v_2,v_3\}$ of *G* lying on *u*.

### Definition

We call an *(oriented) immersion* a pair of maps $\textbf{x}: V\rightarrow \mathbb R^3$ (the immersion) and $\textbf{n}: U\rightarrow \mathbb R^3$ (a normal vector) such that for each face $u=\{v_1,v_2,v_3\}$,

- the points $\textbf{x}_{v_1},\textbf{x}_{v_2},\textbf{x}_{v_3}$ are not collinear. We denote by $\textbf{p}_u\subset \mathbb {R}^3$ their unique common plane.
- $\textbf{n}_u$ is a unit vector normal to $\textbf{p}_u$ oriented in such a way that for any two neighbouring faces $u=\{v_1,v_2,v_3\}$ and $u'=\{v_1,v_2,v'_3\}$, the bases
  $$ (\textbf{x}_{v_2}-\textbf{x}_{v_1},\textbf{x}_{v_3}-\textbf{x}_{v_1},\textbf{n}_u) \text { and } (\textbf{x}_{v_1}-\textbf{x}_{v_2},\textbf{x}_{v'_3}-\textbf{x}_{v_2},\textbf{n}_{u'}) $$
  have the same orientation (are related by a matrix of positive determinant).

See Fig. 1 for an illustration.

For $\textbf{x}_1,\textbf{x}_2,\textbf{x}_3 \in \mathbb R^3$ in general position, we denote by $\textbf{T}[\textbf{x}_1,\textbf{x}_2,\textbf{x}_3]$ the interior of the triangle with vertices $\textbf{x}_1,\textbf{x}_2,\textbf{x}_3$. For $u=\{v_1,v_2,v_3\}$, we define $\textbf{T}^\textbf{x}_u:=\textbf{T}[\textbf{x}_{v_1},\textbf{x}_{v_2},\textbf{x}_{v_3}]$. If we assume that $\textbf{x}_{v_1},\textbf{x}_{v_2},\textbf{x}_{v_3}$ appear on the boundary of $\textbf{T}^\textbf{x}_u$ in the clockwise order around $\textbf{n}_u$ (when looking against the direction of $\textbf{n}_u$, as in Fig. 1), then the orientation condition in the definition above is equivalent to saying that if $u'=\{v_1,v_2,v'_3\}$ is a neighbour face of *u*, then $\textbf{x}_{v_1},\textbf{x}_{v_2},\textbf{x}_{v'_3}$ appear on the boundary of $\textbf{T}^\textbf{x}_{u'}$ in the counterclockwise order around $\textbf{n}_{u'}$ (as in Fig. 1).

**Fig. 2:**
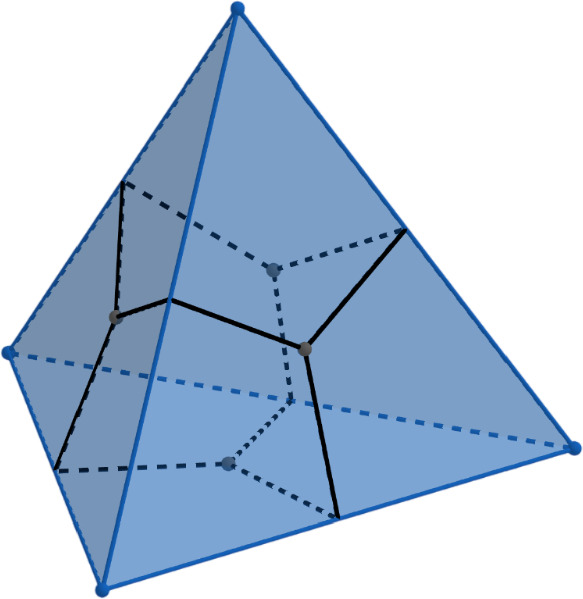
An embedding of a tetrahedron *G* drawn in blue. Its dual tetrahedron $G^*$ is drawn in black

Given an *embedding* $\textbf{x}$ of *G* (by which we mean an oriented immersion with the additional constraint that $\textbf{T}^\textbf{x}_u \cap \textbf{T}^\textbf{x}_{u'} =\emptyset$ for any two distinct faces $u,u'\in U$), Livine and Bonzom [31] introduced the following coupling constants: For two adjacent faces $u=\{v_1,v_2,v_3\}, u'=\{v_1,v_2,v'_3\}\in U$ such that $\textbf{x}_{v_1},\textbf{x}_{v_2},\textbf{x}_{v_3}$ appear on the boundary of $\textbf{T}^\textbf{x}_u$ in the clockwise order around $\textbf{n}_u$ (when looking against the direction of $\textbf{n}_u$), let $\mathcal J=\mathcal J(\textbf{x})$ be defined by

1.1 $$\begin{aligned} \tanh \mathcal J_{\{u,u'\}}=\exp \Big ({ i \frac{\vartheta _{uu'}}{2}}\Big )\sqrt{\tan \frac{\varphi _{uu'}}{2} \tan \frac{\varphi _{u'u}}{2}}, \end{aligned}$$

where $\{u,u'\}\in E^*$, and where

- $\vartheta _{uu'}=\vartheta _{u'u}\in (-\pi ,\pi ]$ is the oriented angle from $\textbf{n}_u$ to $\textbf{n}_{u'}$ (resp. $\textbf{n}_{u'}$ to $\textbf{n}_{u}$) computed clockwise when looking in the direction $\textbf{x}_{v_1}-\textbf{x}_{v_2}$ (resp. $\textbf{x}_{v_2}-\textbf{x}_{v_1}$),
- $\varphi _{uu'}\in (0,\pi )$ (resp. $\varphi _{u'u} \in (0,\pi )$) is the unoriented (*face*) angle between $\textbf{x}_{v_1}-\textbf{x}_{v_3}$ and $\textbf{x}_{v_2}-\textbf{x}_{v_3}$ (resp. between $\textbf{x}_{v_1}-\textbf{x}_{v'_3}$ and $\textbf{x}_{v_2}-\textbf{x}_{v'_3}$), see Fig. 1.

We note that if *u* and $u'$ are coplanar, i.e. $\textbf{p}_u=\textbf{p}_{u'}$, then $\vartheta _{uu'}=0$ and the resulting positive weights were proved to be critical (on infinite tilings of the flat plane) [26].

Our main result is a rigorous proof of the main claim from [31] (see also [17] for numerical evidence) generalised to the setting of immersions (the definition of the weights *y* generalises readily) and with a corrected sign of the $\vartheta$ angles (in [31] the signs of the $\vartheta$ angles were assumed to be constant over the whole embedding, and this was later corrected in [17], which appeared shortly before the current article). In the case of the tetrahedron (see Fig. 2) a proof was given already in [5], and the case of a double pyramid was analytically checked in [17].

### Theorem 1.1

(Livine–Bonzom zeros) Given an immersion $\textbf{x}$ of a finite planar graph *G*, the coupling constants $\mathcal J$ defined in (1.1) satisfy

$$ \mathcal Z_{G^*}(\mathcal J)=0. $$

Our approach (unlike the derivation of [31] that uses a relation to spin networks and loop quantum gravity [4]) makes use of the Kac–Ward matrix [21] (or more precisely its conjugated version studied in [26–29]). Its determinant is equal to the partition function squared, and hence to prove Theorem 1.1 it is enough to construct a vector in its kernel. We do it by a suitable change of coordinates of the eigenvector which reveals a structure of an SU(2) connection on $G^*$. The existence of the null eigenvector turns out to be equivalent to this connection being flat by which we mean that every cycle has trivial holonomy. Due to the classical relation between SU(2) and rotations of $\mathbb R^3$, this has a geometric interpretation in terms of the existence of an immersion of the graph. As a byproduct we establish that the kernel of the Kac–Ward matrix has complex dimension two. We give the details in the remainder of this note.

### Remark 1

As described in [31] the result extends readily (with properly generalised weights) to immersions of graphs *G* where the dual $G^*$ has higher degrees of vertices than three. In that case one requires that the immersion maps the vertices lying around each face of *G* not only to a single plane but moreover to a single circle in $\mathbb R^3$ (which for three vertices is the same condition). If the immersion is locally flat (meaning that the angles $\vartheta$ are zero on a portion of the graph), then this corresponds to circle patterns with the critical weights from [26].

### Remark 2

If $G^*$ has the topology of a torus or a surface of higher genus, then the determinant of an associated Kac–Ward matrix is known to be equal to the square of the partition function of even subgraphs counted with additional signs depending on the homology class of the even subgraph [11, 12, 22]. For example, on the torus, the topologically nontrivial graphs which wind an odd number of times around at least one of the two directions of the torus get an additional minus sign. Our proof shows that with the weights defined in the same way as above, this “twisted” partition function also vanishes. In this case, the additional signs allow for real positive zeros and, as was shown in [12] for the torus, the zero condition is equivalent to criticality of the corresponding doubly periodic Ising model (defined on the universal cover of the finite toroidal graph).

These signs are also those that are missing in the computational check of the formula from [17] for graphs with nontrivial topology.

### Remark 3

As mentioned in [31], the set of zeros defined by (2.1) has real dimension $|E|-1$ (up to global scaling each edge has one real degree of freedom – its length), whereas $|E|-1$ should be the complex dimension (since the set is defined by one complex equation in $|E^*|=|E|$ variables). It is natural to expect that a larger class of zeros will be given by an appropriate three dimensional generalisation of the critical weights of the Ising model on *s-embeddings* defined by Chelkak [7, 8] which in the flat case generalise the critical weights on circle patterns, and which in the case of graphs with toroidal topology parametrise all real positive zeroes of [12] as was shown in [8].

A full description of Ising zeros on planar graphs will be presented in the upcoming work [1].

### Remark 4

Lastly, we want to mention that as the planar Ising model is a special case of the dimer model [6, 13, 14], this work also motivates the question of finding three dimensional geometric descriptions of zeros of the partition function of the dimer model itself (and of its other special cases, e.g. the *uniform spanning tree model*).

## Proof of Theorem 1.1

The proof is organised in sections as follows:

- In Sect. 2.1 we recall the high temperature expansion which is used to rewrite the Ising partition function as a generating function of even subgraphs.
- In Sect. 2.2 we introduce the main tool – the Kac–Ward matrix, whose determinant is the Ising partition function squared.
- The Kac–Ward matrix requires an embedding of the graph in the plane, and in Sect. 2.3 we describe a construction of such an embedding of the dual graph from the given three-dimensional immersion of the primal graph. Along the way, for technical convenience, we modify the dual graph locally without changing the partition function.
- In Sect. 2.4 we show that the associated Kac–Ward matrix on the modified dual graph gives rise to an SU(2) connection on the nonmodified dual graph. The flatness of the connection turns out to be equivalent to the existence of a null eigenvector of the Kac–Ward matrix.
- In Sect. 2.5 we discuss properties of rotations in three dimensions: their Euler angles decomposition and the SU(2) (quaternionic) representation.
- In Sect. 2.6 we check the flatness condition using the definition of the weights $y(\textbf{x})$, and the relation between SU(2) and rotations in three dimensions.

All this means that a null eigenvector (actually a two-dimensional kernel) of the Kac–Ward matrix exists, and as a consequence the partition function of the Ising model vanishes.

### The high-temperature expansion

Let $\mathcal G=(\mathcal V,\mathcal E)$ be again a general finite connected graph (to distinguish from the planar graphs *G* and $G^*$). We call $\omega \subseteq \mathcal E$ an *even subgraph* of $\mathcal G$ if the degree of each $v\in \mathcal V$ in the graph $(\mathcal V,\omega )$ is even. In particular, $\omega =\emptyset$ is an even subgraph. We write $\Omega _\mathcal {G}$ for the set of all even subgraphs of $\mathcal G$, and given a set of weights $x: \mathcal E \rightarrow \mathbb C$, we define the generating function of even subgraphs of $\mathcal G$ by

$$ Z_\mathcal {G}(x)= \sum _{\omega \in \Omega _\mathcal {G}} \prod _{e\in \omega } x_e, $$

where the empty product is defined to be 1. In the case when $\mathcal G$ is trivalent, the even subgraphs are simply collections of disjoint cycles, and then $Z_\mathcal {G}(x)$ is sometimes called the *loop polynomial* of $\mathcal G$ (as in [31]). The next result is classical.

#### Lemma 2.1

(High-temperature expansion). For any finite graph $\mathcal G=(\mathcal V,\mathcal E)$ and all coupling constants $\mathcal J:\mathcal E \rightarrow \mathbb C$ such that $\cosh J_e\ne 0$ for every $e\in \mathcal E$, we have

$$ \mathcal Z_\mathcal {G}(\mathcal J) = Z_\mathcal {G}(x) \times 2^{|\mathcal V|}\prod _{e\in \mathcal E} \cosh \mathcal J_e, \quad \text {where }x_e =\tanh \mathcal J_e. $$

#### Proof

We start by rewriting the Boltzmann factors as

$$ \exp (\mathcal J_{vv'} \sigma _v\sigma _{v'})/\cosh \mathcal J_{vv'}= 1 +\sigma _v\sigma _{v'} \tanh \mathcal J_{vv'} $$

in the definition of the partition function. We subsequently expand the resulting product of sums as a sum of products running over all subsets $\omega$ of $\mathcal E$. The weight of each such subset in this sum is the product over its edges of factors of the form $\sigma _v\sigma _{v'} \tanh \mathcal J_{vv'}$. In the end we notice that the remaining sum over all spin configurations counts each subset $\omega$ with multiplicity $2^{|\mathcal V|}\textbf{1 }\{ \omega \in \Omega _\mathcal {G}\}$. $\square$

To prove Theorem 1.1, it is therefore enough to show that for any oriented immersion $\textbf{x}$ of *G*, we have $Z_{G^*}(y)=0$, where

2.1 $$\begin{aligned} y=y(\textbf{x})= \tanh \mathcal J(\textbf{x}) \end{aligned}$$

with the coupling constants $\mathcal J(\textbf{x})$ given by (1.1).

### The Kac–Ward matrix

Let us now assume that $\mathcal G=(\mathcal V,\mathcal E)$ is as above and moreover *planar*. For two adjacent vertices *v* and $v'$, we will write $\{v,v'\}$ for the corresponding undirected edge, and $vv'$ (resp. $v'v$) for the directed edge from *v* to $v'$ (resp. from $v'$ to *v*). We will also write $\vec {\mathcal {E}}$ for the set of all directed edges of $\mathcal G$.

**Fig. 3:**
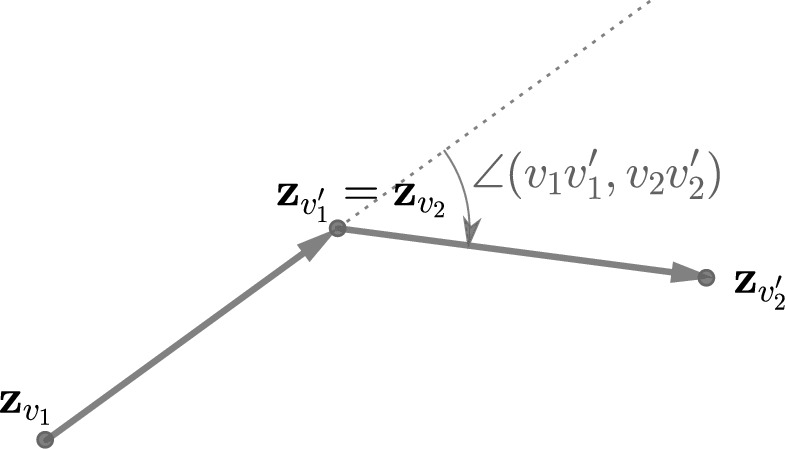
The turning angle $\angle (v_1v_1',v_2v_2')$ (in this case negative)

#### Definition

A *planar embedding* of $\mathcal G$ is an injective map $\textbf{z}: \mathcal V\rightarrow \mathbb C \simeq \mathbb R^2$ such that for any distinct $\{v_1,v'_1\},\{v_2,v'_2\}\in \mathcal E$, the open straight-line segments from $\textbf{z}_{v_1}$ to $\textbf{z}_{v'_1}$ and from $\textbf{z}_{v_2}$ to $\textbf{z}_{v'_2}$ do not intersect.

From now on we fix a planar embedding $\textbf{z}$ of $\mathcal G$. For two directed edges $v_1v_1'$ and $v_2v_2'$, the *turning angle* (under $\textbf{z}$) from $v_1v_1'$ to $v_2v_2'$ is

$$\begin{aligned} \angle (v_1v_1',v_2v_2')= \arg \Big (\frac{\textbf{z}_{v_2'}-\textbf{z}_{v_2}}{\textbf{z}_{v_1'}-\textbf{z}_{v_1}}\Big ) \in (-\pi ,\pi ] \end{aligned}$$

(see Figure 3). Let $\vec x: \vec {\mathcal {E}}\rightarrow \mathbb C$ be weights on the directed edges, and let $x: \mathcal E \rightarrow \mathbb C$ be given by

2.2 $$\begin{aligned} x_{\{v,v'\}}=\vec {x}_{vv'}\vec {x}_{v'v}. \end{aligned}$$

The *(Kac–Ward) transition matrix* $\Lambda _\mathcal {G}(\vec x)$ is the matrix indexed by $\vec {\mathcal {E}}$ given by

$$\begin{aligned} {[}\Lambda _\mathcal {G}(\vec x)]_{v_1v'_1,v_2v'_2} = {\left\{ \begin{array}{ll} \vec {x}_{v'_1v_1} \vec {x}_{v_2v'_2} e^{\frac{i}{2}\angle (v_1v_1',v_2v_2')} &  \text {if } v_1'=v_2 \text { and } v_2'\ne v_1, \\ 0 &  \text {otherwise}. \end{array}\right. } \end{aligned}$$

Note the reversal of the first edge in the weight $\vec {x}_{v'_1v_1}$, and the fact that the matrix assigns nonzero transition weights only to non-backtracking steps. A crucial theorem that goes back to the work of Kac and Ward [21] is the following (for streamlined proofs, see e.g. [9, 29]).

#### Theorem 2.2

For any planar embedding of a planar graph $\mathcal G=(\mathcal V,\mathcal E)$, and any choice of weights satisfying (2.2), we have

$$\begin{aligned} Z_\mathcal {G}^2(x)={\det }(\textrm{Id}-\Lambda _\mathcal {G}(\vec x)), \end{aligned}$$

where $\textrm{Id}$ is the identity matrix on $\vec {\mathcal {E}}$.

In particular the determinant is independent of the choice of the embedding $\textbf{z}$, and it depends on $\vec x$ only through *x*. It immediately follows that $Z_\mathcal {G}(x)=0$ if and only if there exists a nontrivial $\rho : \vec {\mathcal {E}} \rightarrow \mathbb C$ such that $\Lambda _\mathcal {G}(\vec x) \rho =\rho$. The rest of this note is devoted to the construction of such a vector in the (slightly modified) setting of Theorem 1.1.

### A graph modification and planar decompositions

The first observation is that given a finite graph $\mathcal G=(\mathcal V,\mathcal E)$ and weights $x:\mathcal E\rightarrow \mathbb C$, one can subdivide each edge $e\in \mathcal E$ into a number of subedges $e_i$ connected in a series, and then assign weights $x_{e_i}$ in such a way that $x_e=\prod _{i}x_{e_i}$, and the resulting weighted graph will have the same generating function of even subgraphs as $\mathcal G$.

Recall that $G^*$ is the graph on which the Ising model is defined, and it is the dual of *G*, which is immersed in $\mathbb R^3$. We apply the procedure described above to $G^*$ and divide each edge into three subedges. More precisely, each edge $\{u,u'\}\in E^*$ is replaced by three edges $\{u,u_1\}, \{u_1,u_2\}, \{u_2,u'\}$, where we moreover identify $u_1$ with the directed edge $uu'$, and $u_2$ with the edge $u'u$. We denote the resulting graph by $G^\dagger =( U^\dagger , E^\dagger )$ (where $U^\dagger \simeq U\cup \vec E^*$) and we extend the weights *y* defined in (2.1) to $E^\dagger$ by setting

$$\begin{aligned} y_{\{u,uu'\}}= \sqrt{\tan \Big (\frac{\varphi _{uu'}}{2}\Big )}, \quad y_{\{uu',u'u\}}= \exp \Big ({ i \frac{\vartheta _{uu'}}{2}}\Big ), \quad y_{\{u'u,u'\}}= \sqrt{\tan \Big (\frac{\varphi _{u'u}}{2}\Big )}. \end{aligned}$$

It follows that $Z_{G^*}(y)= Z_{ G^\dagger }(y)$, which will be crucial in the remainder of this note.

**Fig. 4:**
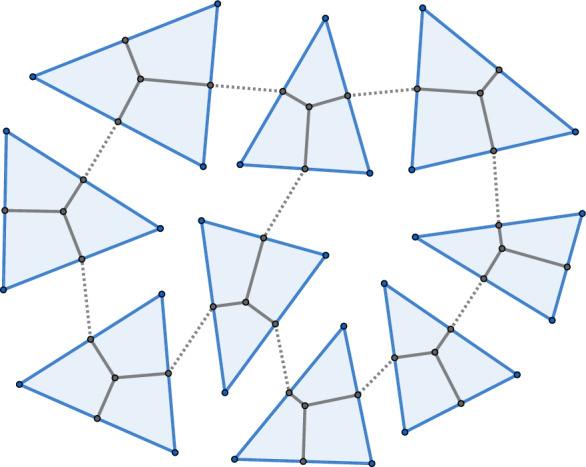
A piece of a planar decomposition of an immersion

Recall that $\textbf{x}$ is an oriented immersion of *G* into $\mathbb R^3$. It will be useful to extend $\textbf{x}$ to the set of faces *U* of *G* by assigning to each $u=\{v_1,v_2,v_3\}$ a chosen point $\textbf{x}_u$ inside the triangle $\textbf{T}^\textbf{x}_u$ with the property that its projections on the sides of the triangle lie inside the sides themselves (for example, the centre of the inscribed circle). The precise location of the point is a technical choice, and the only important feature is the angles between the projections. They are expressed through the angles of the triangle itself and their exact value is used in the proof of Lemma 2.3.

A *corner* of $G^*$ is a pair *uv* composed of a face $u\in U$ and an incident vertex $v\in V$. We denote by *C* the set of all corners of $G^*$. In what follows we identify $\mathbb C\simeq \mathbb R^2$ with the subspace of $\mathbb R^3$ spanned by the first two vectors in the standard basis $(e_1,e_2,e_3)$ of $\mathbb R^3$. We also think of triangles embedded in $\mathbb C$ as oriented in $\mathbb R^3$, where the orientation comes from the natural orientation of $\mathbb R^3$. In particular the normal to a triangle points in the (positive) direction of $e_3$.

#### Definition

A *planar decomposition* of an immersion $\textbf{x}$ consists of

- a map $\textbf{z}: C\rightarrow \mathbb C$ such that for each face $u=\{v_1,v_2,v_3\}$, the triangle $\textbf{T}^\textbf{z}_u:=\textbf{T}[\textbf{z}_{uv_1},\textbf{z}_{uv_2}, \textbf{z}_{uv_3}] \subset \mathbb C$ is isometric to $\textbf{T}^\textbf{x}_u=\textbf{T}[\textbf{x}_{v_1},\textbf{x}_{v_2}, \textbf{x}_{v_3}] \subset \mathbb R^3$ by an orientation-preserving isometry of $\mathbb R^3$ (here we use the fact that $\textbf{T}^\textbf{x}_u$ comes with an orientation induced by the immersion $\textbf{x}$, $\textbf{T}^\textbf{z}_u$ comes with the orientation described above, and we want to avoid “flipping the triangles upside-down”), and moreover $\textbf{T}^\textbf{z}_u\cap \textbf{T}^\textbf{z}_{u'}=\emptyset$ for each pair of distinct $u, u'\in U$. We denote by $\textbf{z}_u$ the image of $\textbf{x}_u$ under the corresponding isometry.
- A collection of piecewise linear and pairwise nonintersecting paths in $\mathbb C \setminus \bigcup _{u\in U} \textbf{T}^\textbf{z}_u$ that connect the projection of $\textbf{z}_u$ on the line segment $[\textbf{z}_{uv_1},\textbf{z}_{uv_2}]$ with the projection of $\textbf{z}_{u'}$ on the line segment $[\textbf{z}_{u'v_1},\textbf{z}_{u'v_2}]$ for each pair of neighbouring faces $u=\{v_1,v_2,v_3\}$, $u'=\{v_1,v_2,v'_3\}$.

**Fig. 5:**
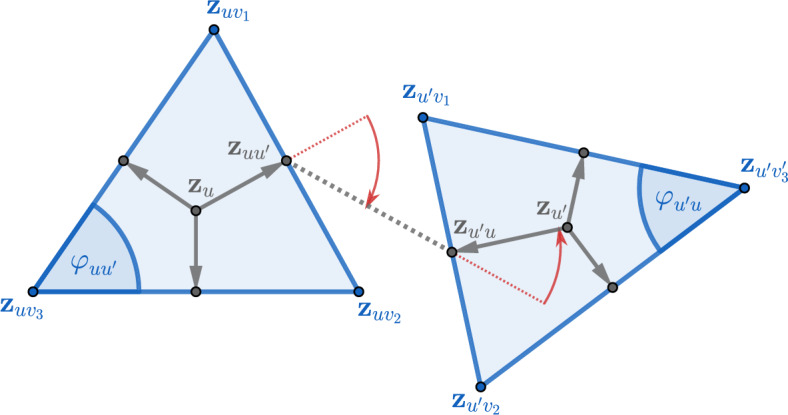
Planar decomposition in the vicinity of two faces *u* and $u'$. The angle $\alpha _{uu'}$ is equal to the sum of the two oriented red angles. Also $\beta _{uu'}=\arg (\textbf{z}_{uv_1}-\textbf{z}_{uv_2})$ and $\beta _{u'u}=\arg (\textbf{z}_{u'v_2}-\textbf{z}_{u'v_1})$. The reversal of the roles of $v_1$ and $v_2$ in these two angles arises from the clockwise order of vertices around the face used in their definition

See Figs. 4 and 5 for an illustration.

We note that there always exists a planar decomposition of an immersion $\textbf{x}$. Indeed, it is enough to start with a planar embedding of $G^*$ so that the lengths of the edges are much larger than the lengths of the edges of the triangles under $\textbf{x}$. One can then draw the corresponding triangles over the vertices of $G^*$, and finally replace the long edges by piecewise linear paths. From now on, for simplicity of the notation, we assume that these paths consist of single edges (as shown in Fig. 4). The general case follows similarly.

We will extend $\textbf{z}$ to $\vec E^*\cup U$ by defining $\textbf{z}_{uu'}$ (resp. $\textbf{z}_{uu'}$) to be the projection of $\textbf{z}_u$ on the line segment $[\textbf{z}_{uv_1},\textbf{z}_{uv_2}]$ (resp. of $\textbf{z}_{u'}$ on the line segment $[\textbf{z}_{u'v_1},\textbf{z}_{u'v_2}]$), and by taking $\textbf{z}_u$ as defined above.

This means that a planar decomposition of an immersion of *G* gives rise to a planar embedding (as defined in Sect. 2.2) of the modified graph $G^\dagger =( U^\dagger , E^\dagger )$ (where $U^\dagger =U\cup \vec E^*$). We will work with the Kac–Ward transition matrix associated with this planar embedding and with the Ising weights *y* as defined above. The corresponding Kac–Ward weights on the directed edges $\vec E^\dagger$ are chosen as follows

2.3 $$\begin{aligned} \vec y_{u(uu')}=y_{\{u,(uu')\}},\ \vec y_{(uu')u}=1, \ \vec y_{(uu')(u'u)}=\vec y_{(u'u)(uu')}= \exp \Big ({ i \frac{\vartheta _{uu'}}{4}}\Big ), \end{aligned}$$

where we write $(uu')$ for a directed edge $uu'\in U^\dagger$ to avoid confusion. It is clear that $\vec y_{ww'}\vec y_{w'w}= y_{\{w,w'\}}$ for all neighbours $w,w'\in U^\dagger$, where *y* is as above. Hence, to prove Theorem 1.1 it is enough to construct an vector $\rho : \vec E^\dagger \rightarrow \mathbb C$ such that $\Lambda _{G^\dagger }(\vec y)\rho =\rho$.

### A change of coordinates and an SU(2) connection

From now on we write $\Lambda =\Lambda _{G^\dagger }(\vec y)$. Following [26, 27], for $w\in U^\dagger$, let $\text {Out}_w = \{ww':ww' \in \vec E^\dagger \}$, and let *J* be the involutive automorphism of $\mathbb {C}^{\vec E^\dagger }$ induced by the map $ww' \mapsto w'w$. As was noted in [27], and is easily seen from the definition of the Kac–Ward transition matrix, the matrix ${\tilde{\Lambda }}:=J \Lambda$ is Hermitian, block-diagonal with blocks ${\tilde{\Lambda }}^w$, $w\in U^\dagger$, acting on the linear subspace indexed by $\text {Out}_w$, and given by

2.4 $$\begin{aligned} {\tilde{\Lambda }}^w_{ww', ww''} = {\left\{ \begin{array}{ll} \vec {y}_{ww'} \vec {y}_{ww''} e^{\frac{i}{2}\angle (w'w, ww'')} &  \text {if } w' \ne w'' , \\ 0 &  \text {otherwise}. \end{array}\right. } \end{aligned}$$

Note that ${\tilde{\Lambda }}^w$ is two dimensional if $w\in \vec E^*$ and three dimensional if $w\in U$. A direct computation shows the following.

#### Lemma 2.3

When $u\in U$, the spectrum of ${\tilde{\Lambda }}^u$ is $\{-1,0,1\}$, and moreover

$$\begin{aligned} {\tilde{\Lambda }}^u \rho ^{u,+}= \rho ^{u,+} \quad \text { and } \quad {\tilde{\Lambda }}^u \rho ^{u,-}= -\rho ^{u,-}, \end{aligned}$$

with $\rho ^{u,\pm }\in \mathbb C^{\text {Out}_u}$ given by

$$\begin{aligned} \rho ^{u,+}_{u(uu')} =\kappa _{uu'} \quad \text { and } \quad \rho ^{u,-}_{u(uu')} =\kappa _{uu'}e^{-i\beta _{uu'}}, \end{aligned}$$

where

$$\begin{aligned} \kappa ^2_{uu'}= {|\textbf{z}_{uv_1}-\textbf{z}_{uv_2}|} \quad \text { and } \quad e^{i\beta _{uu'}}=\frac{\textbf{z}_{uv_1}-\textbf{z}_{uv_2}}{{|\textbf{z}_{uv_1}-\textbf{z}_{uv_2}|}}. \end{aligned}$$

Here $u(uu')\in \text {Out}_u$, and $u=\{v_1,v_2,v_3\}$ are listed clockwise and $u'=\{v_1,v_2,v'_3\}$ are listed counterclockwise (see Fig. 5).

For the proof, see Appendix A.

For future reference, we note that $\kappa ^2_{uu'}=\kappa ^2_{u'u}=|\textbf{x}_{v_1}-\textbf{x}_{v_2}|$, and elementary geometry implies that

2.5 $$\begin{aligned} e^{i(\beta _{uu'}-\beta _{u'u})}=\frac{\textbf{z}_{uv_1}-\textbf{z}_{uv_2}}{\textbf{z}_{u'v_2}-\textbf{z}_{u'v_1}}=-e^{-i\alpha _{uu'}}, \end{aligned}$$

where

$$\begin{aligned} \alpha _{uu'} = \angle (uu_1,u_1u_2)+\angle (u_1u_2,u_2u'), \end{aligned}$$

with $u_1=uu',u_2=u'u\in U^\dagger$ (see Fig. 5).

We now assume that there exists a vector $\rho : \vec E^\dagger \rightarrow \mathbb C$ such that $\Lambda \rho =\rho$, and hence ${\tilde{\Lambda }}\rho =J\Lambda \rho =J\rho$. Since *J* is an isometry for the Euclidean norm $\Vert \cdot \Vert$ on $\mathbb {C}^{\vec E^\dagger }$, and since ${\tilde{\Lambda }}$ is block diagonal, we can write

$$\begin{aligned} \sum _{w\in U^\dagger } \Vert \rho ^w\Vert ^2=\Vert \rho \Vert ^2= \Vert {\tilde{\Lambda }}\rho \Vert ^2=\sum _{w\in U^\dagger } \Vert {\tilde{\Lambda }}^w\rho ^w\Vert ^2, \end{aligned}$$

where $\rho ^w$ is the restriction of $\rho$ to the subspace indexed by $\text {Out}_w$, and where $\Vert \cdot \Vert$ is the Euclidean operator norm. Since the blocks ${\tilde{\Lambda }}^w$ are Hermitian their operator norm is equal to the spectral radius. If $w\in U$, then by Lemma 2.3, the spectral radius of ${\tilde{\Lambda }}^w$ is one. On the other hand if $w\in \vec E^*$, then by (2.4) and (2.3), ${\tilde{\Lambda }}^w$ is a $2\times 2$ antidiagonal matrix with entries of modulus one, and hence also of spectral radius one. We conclude that if $\Lambda \rho =\rho$, then for all $u\in U$, $\Vert \rho ^u\Vert =\Vert {\tilde{\Lambda }} \rho ^u\Vert$ and hence by Lemma 2.3,

$$\begin{aligned} \rho _{uw}=\rho ^u_{uw} = \xi _u^{+} \rho ^{u,+}_{uw}+ \xi _u^{-} \rho ^{u,-}_{uw} \end{aligned}$$

for some $\xi _u^{\pm }\in \mathbb C$. We therefore also have

$$\begin{aligned} \rho _{wu}=\Lambda \rho _{wu} =J{\tilde{\Lambda }}^u \rho ^u_{wu} =J(\xi _u^{+} \rho ^{u,+}_{wu}- \xi _u^{-} \rho ^{u,-}_{wu})=\xi _u^{+} \rho ^{u,+}_{uw}- \xi _u^{-} \rho ^{u,-}_{uw}. \end{aligned}$$

This in particular means that the new coordinates $\xi _u=(\xi _u^{+},\xi _u^{-})$, $u\in U$, fully determine the eigenvector $\rho$ around each $u\in U$, which in turn determines the full eigenvector.

We now want to see how $\xi ^u$ and $\xi ^{u'}$ should be related to each other for neighbouring faces ${u,u'\in U}$. To this end, we note that when propagating the value $\rho _{(u'u)u'}$ to $\rho _{u(uu')}$ (resp. $\rho _{(uu')u}$ to $\rho _{u'(u'u)}$) by applying $\Lambda$ (left application of $\Lambda$ moves us backwards along the directed path from *u* to $u'$), one picks up the phase $e^{\tfrac{i}{2}(\vartheta _{uu'}+\alpha _{uu'})}$ (resp. $e^{\tfrac{i}{2}(\vartheta _{uu'}-\alpha _{uu'})}$), and hence

$$\begin{aligned} \rho _{u(uu')} = \rho _{(u'u)u'}e^{\tfrac{i}{2}(\vartheta _{uu'}+\alpha _{uu'})} \quad \text { and } \quad \rho _{u'(u'u)} = \rho _{(uu')u}e^{\tfrac{i}{2}(\vartheta _{uu'}-\alpha _{uu'})}. \end{aligned}$$

This together with the two displayed equations above and the explicit form of the eigenvectors from Lemma 2.3 give (after factoring out $\kappa _{uu'}$ from both equations)

$$\begin{aligned} \xi _{u'}^+-\xi _{u'}^-e^{-i\beta _{u'u}}&=(\xi _{u}^++\xi _u^-e^{-i\beta _{uu'}})e^{\tfrac{i}{2}(-\vartheta _{uu'}-\alpha _{uu'})},\\ \xi _{u'}^{+}+\xi _{u'}^{-}e^{-i\beta _{u'u}}&=(\xi _u^{+}-\xi _u^{-}e^{-i\beta _{uu'}})e^{\tfrac{i}{2}(\vartheta _{uu'}-\alpha _{uu'})}, \\ \end{aligned}$$

which in matrix form reads

$$ \xi _{u'}= \xi _{u} \Upsilon _{uu'}, $$

where

$$\begin{aligned} \Upsilon _{uu'}&=e^{-\frac{i}{2} \alpha _{uu'}}\begin{pmatrix} \cos \frac{\vartheta _{uu'}}{2} &  ie^{ i\beta _{u'u}}\sin \frac{\vartheta _{uu'}}{2} \\ -ie^{- i\beta _{uu'}}\sin \tfrac{\vartheta _{uu'}}{2} &  -e^{i(\beta _{u'u}-\beta _{uu'}) } \cos \tfrac{\vartheta _{uu'}}{2} \end{pmatrix} \\&=\begin{pmatrix}e^{-\frac{i}{2} \alpha _{uu'}} \cos \frac{\vartheta _{uu'}}{2} &  -ie^{\frac{i}{2} \alpha _{uu'}+i\beta _{uu'}}\sin \frac{\vartheta _{uu'}}{2} \\ -ie^{-\frac{i}{2} \alpha _{uu'}- i\beta _{uu'}}\sin \tfrac{\vartheta _{uu'}}{2} &  e^{\frac{i}{2} \alpha _{uu'}} \cos \tfrac{\vartheta _{uu'}}{2} \end{pmatrix}. \end{aligned}$$

In the second identity we used (2.5). On can see that $\Upsilon _{uu'}\in \text {SU}(2)$ as it is of the form

2.6 $$\begin{aligned} \begin{pmatrix} a +b i &  c+di \\ -c+di &  a-bi \end{pmatrix} \end{aligned}$$

with $$a,b,c,d\in \mathbb R$$ and $$a^2+b^2+c^2+d^2=1$$.

By construction, $\Upsilon _{u'u}=\Upsilon _{uu'}^{-1}$, and therefore the matrices $\Upsilon _{uu'}$, $uu'\in \vec E^*$, form an $\text {SU}(2)$-connection $\Upsilon$ on $G^*$.

We say that a connection is *flat*, if the product of its elements along any closed directed loop in $G^*$ (called the *holonomy* of the loop) is the identity matrix.

#### Lemma 2.4

A non-zero vector $\rho : \vec E^\dagger \rightarrow \mathbb C$ such that $\Lambda \rho =\rho$ exists if and only if $\Upsilon$ is flat.

#### Proof

If such $\rho$ exists, then the coordinates $\xi _u$ are well defined, and hence $\Upsilon$ is flat by definition. On the other hand, if $\Upsilon$ is flat, then one can consistently recover all $\xi _u$ from any fixed $\xi _{u_0}$ (since the graph is connected), and then construct $\rho$ locally around every $u\in U$. $\square$

To prove Theorem 1.1 it is therefore enough to show that $\Upsilon$ is flat.

### Euler angles and rotations in 3D

To this end, we will need a geometric interpretation of $\text {SU}(2)$ matrices. Recall that the *Euler angles* $\phi$ and $\psi$ associated with the matrix in (2.6) are given by

$$\begin{aligned} \psi + \phi =2 \arg (a+bi) \qquad \text {and} \qquad \psi -\phi = 2\arg (c+di). \end{aligned}$$

A computation yields that for $\Upsilon _{uu'}$, the Euler angles are

2.7 $$\begin{aligned} \psi _{uu'}=\beta _{uu'}-\frac{\pi }{2} \qquad \text {and} \qquad \phi _{uu'} =\frac{\pi }{2}- \alpha _{uu'}-\beta _{uu'}=\frac{3\pi }{2}-\beta _{u'u}, \end{aligned}$$

where we again used (2.5). Hence we can decompose into elemental $\text {SU}(2)$ matrices:

2.8 $$\begin{aligned} \Upsilon _{uu'}&=\begin{pmatrix} e^{\frac{i}{2}(\psi _{uu'}+\phi _{uu'})}\cos \frac{\vartheta _{uu'}}{2} &  e^{\frac{i}{2}(\psi _{uu'}-\phi _{uu'})}\sin \frac{\vartheta _{uu'}}{2} \\ -e^{-\frac{i}{2}(\psi _{uu'}-\phi _{uu'})}\sin \tfrac{\vartheta _{uu'}}{2} &  e^{-\frac{i}{2}(\psi _{uu'}+\phi _{uu'})}\cos \tfrac{\vartheta _{uu'}}{2} \end{pmatrix}\nonumber \\&= \begin{pmatrix} e^{i \frac{\psi _{uu'}}{2}}&  0 \\ 0 &  e^{-i \frac{\psi _{uu'}}{2}} \end{pmatrix} \begin{pmatrix} \cos \frac{\vartheta _{uu'}}{2} &  \sin \frac{\vartheta _{uu'}}{2} \\ -\sin \tfrac{\vartheta _{uu'}}{2} &  \cos \tfrac{\vartheta _{uu'}}{2} \end{pmatrix} \begin{pmatrix} e^{i \frac{\phi _{uu'}}{2}}&  0 \\ 0 &  e^{-i \frac{\phi _{uu'}}{2}} \end{pmatrix} \nonumber \\&=:\Upsilon _z(\psi _{uu'})\Upsilon _x(\vartheta _{uu'})\Upsilon _z(\phi _{uu'}). \end{aligned}$$

The notation $\Upsilon _x(\gamma )$ and $\Upsilon _z(\gamma )$ refers to rotations around the *x* and *z* axis of $\mathbb R^3$ by an angle $\gamma$. To justify it, we need to recall the connection between $\text {SU}(2)$ and $\text {SO}(3)$ – the group of rotations of $\mathbb R^3$. We chose to use the representation of $\text {SU}(2)$ as the unit *quaternions*, i.e. each matrix as in (2.6) is associated with the quaternion $q=a+b\textbf{i}+c\textbf{j}+d\textbf{k}$. We note that this representation is not strictly necessary for the understanding of the rest of the proof but we choose to recall it as a convenient tool for thinking about rotations. We refer the reader to [19] for an exhaustive account of this relation. With the standard rules of quaternion multiplication ($\textbf{i}^2=\textbf{j}^2=\textbf{k}^2=\textbf{i}\textbf{j}\textbf{k}=-1$, $\textbf{i}\textbf{j}=\textbf{k}$, $\textbf{j}\textbf{k}=\textbf{i}$ and $\textbf{k}\textbf{i}=\textbf{j}$) the map is a group isomorphism between $\text {SU}(2)$ and quaternions $q=a+b\textbf{i}+c\textbf{j}+d\textbf{k}$ of unit norm, i.e. such that $\Vert q\Vert ^2=a^2+b^2+c^2+d^2=1$. Recall that ${\bar{q}}= a-b\textbf{i}-c\textbf{j}-d\textbf{k}$ and $q{\bar{q}}={\bar{q}} q =\Vert q\Vert ^2$. We call a quaternion *q* *pure* if $\operatorname {Re}q:=\tfrac{1}{2}(q+{\bar{q}})=0$. The set of pure quaternions can be identified with $\mathbb R^3$ by the map $b\textbf{i}+c\textbf{j}+d\textbf{k}\mapsto (d,c,b)$ (we chose this particular order for convenience so that $\textbf{i}$ corresponds to the *z* coordinate and $\textbf{k}$ to the *x* coordinate in $\mathbb R^3$). Each unit quaternion *q* can be written as

$$\begin{aligned} q= \cos \frac{\gamma }{2}+p \sin \frac{\gamma }{2} \end{aligned}$$

where *p* is a pure unit quaternion. It is classical that if $p_0$ is another pure quaternion, then

$$\begin{aligned} p'_0=q p_0 q^{-1} \end{aligned}$$

is the pure quaternion resulting from the rotation of $p_0$ around the axis given by *p* by an angle of $\gamma$ (note the doubling of the angle). Here the rotation is clockwise when looking in the direction of *p*. This map $q \mapsto R_p(\gamma )$, where $R_p(\gamma )\in \text {SO}(3)$ is the corresponding rotation matrix, is a 2-to-1 group homomorphism from $\text {SU}(2)$ to $\text {SO}(3)$. The multiplicity of this map comes from the fact that $-q$ gives rise to the same rotation as *q*. This will be enough knowledge about $\text {SU}(2)$ to finish the proof of Theorem 1.1. In particular, this justifies the notation from (2.8).

**Fig. 6:**
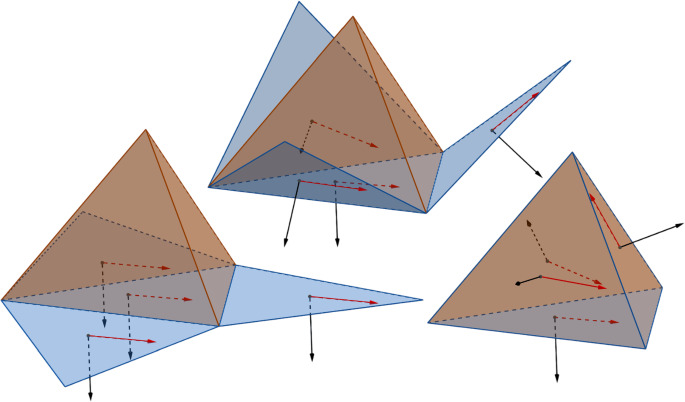
From left to right: the folding of a net (a special case of a flat decomposition) of a tetrahedron together with a constant vector field (red vectors) on the faces into a framed immersion of the tetrahedron. To match our conventions, the bottom plane on which the net lies should be seen upside down so that the black vectors point upwards. The red vectors in the plane are in our case taken to be *i*, and they get mapped to the $\textbf{r}_u$ vectors by the folding

Before proceeding we still need to recall one classical fact, this time about rotations themselves. Namely, a sequence of rotations around the coordinate axes of a fixed reference frame (*extrinsic rotations*) is equivalent to the reverse sequence of the same rotations but this time taken around the corresponding axes of the moving (rotating) frame (*intrinsic rotations*). This follows from the fact that the change of basis operation is realised by matrix conjugation (here the change of basis matrices are the rotations themselves). By (2.8), $\Upsilon _{uu'}$ corresponds to the rotation $R_{u,u'}$ resulting from the composition $R_z(\psi _{uu'})R_x(\vartheta _{uu'})R_z(\phi _{uu'})$, where *x* (resp. *z*) stands for $e_1$ (resp. $e_3$) in the standard frame $(e_1,e_2,e_3)$. Assuming the initial frame agrees with the standard frame, this corresponds to the composition

2.9 $$\begin{aligned} R_{u,u'}=R_{z''}(\phi _{uu'})R_{x'}(\vartheta _{uu'})R_z(\psi _{uu'}), \end{aligned}$$

where $x'=R_z(\psi _{uu'})e_1$ and $z''=R_{x'}(\vartheta _{uu'})R_z(\psi _{uu'})e_3$, are the *x* and *z* axes of the rotating frame after one and two rotations respectively. This interpretation will be very convenient to show that the connection $\{\Upsilon _{uu'}\}_{uu'\in \vec G^*}$ is flat. Before doing this, we will need one more definition.

### Framed immersions and flatness of the connection

Recall that for a face $u=\{v_1,v_2,v_3\}$, $\textbf{p}_u$ is the plane on which $\textbf{x}_{v_1},\textbf{x}_{v_2},\textbf{x}_{v_3}$ lie.

#### Definition

A *framed immersion* is an oriented immersion $\textbf{x}$ together with a choice of a unit vector $\textbf{r}_u \in \mathbb R^3$ parallel to $\textbf{p}_u$ for each face $u\in U$.

See Fig 6 for an illustration.

We now describe how to obtain a framed immersion associated with a planar decomposition. Before reading the formal definition the reader may look at Fig. 6 which readily illustrates this procedure in the case of the tetrahedron. To be more formal, consider a planar decomposition $\textbf{z}$ of an immersion $\textbf{x}$. For each $u=\{v_1,v_2,v_3\}\in U$, let $\iota _u$ be the isometry of $\mathbb R^3$ that maps $\textbf{T}^\textbf{z}_u$ to $\textbf{T}^\textbf{x}_u$, and such that $\iota _u(\textbf{z}_u+(0,0,1))-\iota _u(\textbf{z}_u)=\textbf{n}_u$. Here we still think of $\mathbb C\simeq \mathbb R^2$ as the *x*-*y* plane of $\mathbb R^3$. Then we simply define $\textbf{r}_u= \iota _u(i)$, where the complex unit *i* is identified with $(0,1,0)\in \mathbb R^3$.

#### Proposition 2.5

The connection $\Upsilon$ given by (2.8) is flat.

#### Proof

**Fig. 7:**
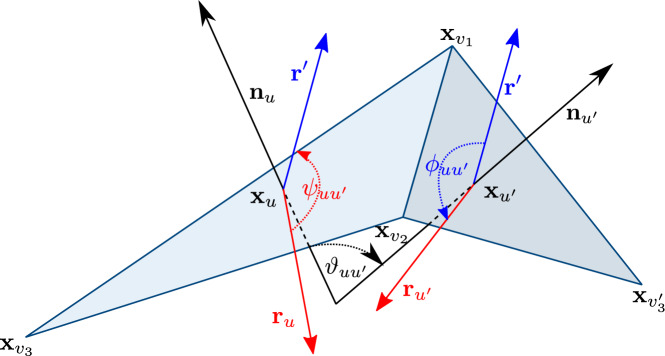
Two adjacent faces in a framed immersion obtained from a planar decomposition. The composition of three intrinsic rotations $R_{x''}(\phi _{uu'})R_{z'}(\vartheta _{uu'})R_x(\psi _{uu'})$ rotates the frame $(\textbf{n}_u,\textbf{r}_u)$ to the frame $(\textbf{n}_{u'},\textbf{r}_{u'})$ by the sequence of transformations $(\textbf{n}_u,\textbf{r}_u)\rightarrow (\textbf{n}_u,\textbf{r}')\rightarrow (\textbf{n}_{u'},\textbf{r}')\rightarrow (\textbf{n}_{u'},\textbf{r}_{u'})$. The intermediate vector $\textbf{r}'_u$ is equal to $(\textbf{x}_{v_1}-\textbf{x}_{v_2})/|\textbf{x}_{v_1}-\textbf{x}_{v_2}|$

We will denote by $F_u$ the rotation from the standard frame $(e_1,e_2,e_3)$ to the positively oriented frame $(\textbf{r}_u, \textbf{n}_u\times \textbf{r}_u, \textbf{n}_u)$. We will identify $F_u$, through its matrix representation, with the frame itself written in column form. In this setup we now make the following claims (that are illustrated in Fig. 7). Applying $F^{-1}_u$ first to the immersion we may assume the frame at *u* is the standard one. We will only track the two axes $(\textbf{r}_u,\textbf{n}_u)$ as the third one is determined. We now consider the sequence of intrinsic rotations from (2.9) that define the rotation $R_{u,u'}$. We have that

- $R_z(\psi _{uu'})=R_{\textbf{n}_u}(\psi _{uu'})$ maps $(\textbf{r}_u,\textbf{n}_u)$ to $(\textbf{r}',\textbf{n}_u)$, where
  $$ \textbf{r}' =\frac{\textbf{x}_{v_1}-\textbf{x}_{v_2}}{|\textbf{x}_{v_1}-\textbf{x}_{v_2}|}. $$
  Indeed
  $$\begin{aligned} \psi _{uu'}&= \beta _{uu'}-\frac{\pi }{2} =\arg \Big (\frac{\textbf{z}_{uv_1}-\textbf{z}_{uv_2}}{i}\Big ) \end{aligned}$$
  is the rotation angle from *i* to $\textbf{z}_{uv_1}-\textbf{z}_{uv_2}$, which by construction is the same as the rotation angle from $\textbf{r}_u$ to $\textbf{r}'$.
- $R_{x'}(\vartheta _{uu'})=R_{\textbf{r}'}(\vartheta _{uu'})=R_{\textbf{x}_{v_1}-\textbf{x}_{v_2}}(\vartheta _{uu'})$ maps $(\textbf{r}',\textbf{n}_u)$ to $(\textbf{r}',\textbf{n}_{u'})$ by the definition of the angle $\vartheta _{uu'}$.
- $R_{z''}(\phi _{uu'})=R_{\textbf{n}_{u'}}(\phi _{uu'})$ maps $(\textbf{r}'_u,\textbf{n}_{u'})$ to $(\textbf{r}_{u'}, \textbf{n}_{u'})$. Indeed
  $$\begin{aligned} \phi _{uu'}&=\pi +\frac{\pi }{2}-\beta _{u'u} =\pi +\arg \Big (\frac{i}{\textbf{z}_{u'v_2}-\textbf{z}_{u'v_1}}\Big ) =\arg \Big (\frac{i}{\textbf{z}_{u'v_1}-\textbf{z}_{u'v_2}}\Big ) \end{aligned}$$
  is the rotation angle from $\textbf{z}_{u'v_1}-\textbf{z}_{u'v_2}$ to *i*, which by construction is the same as the rotation angle from $\textbf{r}'$ to $\textbf{r}_{u'}$.

Accounting for the initial rotation $F^{-1}_u$ from the original frame at *u* to the standard frame (identified with the identity matrix $\textrm{Id}$), we obtain that $R_{u,u'} =R_{u,u'} \textrm{Id}=F^{-1}_u F_{u'}$, and equivalently $F_uR_{u,u'} =F_{u'}$. This means that $R_{u,u'}$ maps $F_u$ to $F_{u'}$ by multiplication on the right, and hence $\Upsilon _{uu'}$ acts by rotating the frame at *u* to the frame at $u'$ also through multiplication on the right. Hence, if we follow a closed loop starting at *u* and apply $\Upsilon$ iteratively, the frame at *u* will be mapped to itself. However, since the homomorphism from $\text {SU}(2)$ to $\text {SO}(3)$ is 2-to-1 it is still possible that the holonomy of the this loop is equal to minus the identity matrix (which also corresponds to the trivial rotation).

We will now argue why this is not the case. It is enough to show that the holonomy of elementary loops defining the faces of $G^*$ is the identity (loops going around a single vertex *v* of *G*). To this end note that in the flat case, i.e. when all the $\vartheta$ angles along the loop are zero, a planar decomposition can be chosen so that the paths between $uu'$ and $u'u$ have zero winding $\alpha _{uu'}$ (indeed they are trivial paths if $\textbf{z}_{uu'}=\textbf{z}_{u'u}$, or can be chosen to make two opposite right turns). In that case by (2.5) we have $e^{i(\beta _{uu'}-\beta _{u'u})}=-1$, and hence $e^{i(\psi _{uu'}+\phi _{u'u})}=1$, which in turn implies that $\Upsilon _{uu'}=\textrm{Id}$ for all edges $uu'$ along the loop. In particular the product over the loop is the identity. One can now modify the picture by continuously changing the angles $\vartheta$ and $\varphi$ around *v*. The holonomy along the loop is clearly a continuous function of all the angles (as seen in (2.8)), and hence it cannot discontinuously change from $\textrm{Id}$ to $-\textrm{Id}$. $\square$

This ends the proof of Theorem 1.1.

#### Remark 5

As mentioned in the introduction, from the proof it follows that any choice of $\xi _{u_0}$ for some fixed $u_0\in U$ gives rise to an eigenvector of $\Lambda$ with eigenvalue one (by propagating $\xi _{u_0}$ to any other face by applications of $\Upsilon$). It also follows that any such eigenvector gives rise to such $\xi _{u_0}$, and hence the kernel of $\textrm{Id}-\Lambda$ has two complex dimensions corresponding to the two coordinates of $\xi _{u_0}$.

#### Remark 6

It is known from [9] that the Kac–Ward matrix is equivalent to an antisymmetric matrix of even size, and as such its kernel has even dimension due to the Youla decomposition [38]. Here we have shown that for the Livine-Bonzom weights, this dimension is always two. We thank the anonymous referee for this remark.

#### Remark 7

The collection $\{\xi _{u}\}_{u\in U}$ can be seen as a field of two-component *spinors* acted upon by the $\text {SU}(2)$ connection $\{\Upsilon _{uu'}\}_{uu'\in \vec E^*}$. Each such field represents a null eigenvector of $\textrm{Id}-\Lambda$.

We note that spinors assigned to the vertices of the graph appear also in the spin network picture of [4] but a precise connection with our setup is unclear.

Moreover, in loop quantum gravity physical states are supported on the moduli space of flat $\text {SU}(2)$ connections modulo gauge and diffeomorphisms [2, 3, 16, 18, 34]. However, at this point we do not see a direct relation with the flat connection appearing in our work.

## Figures and Tables

**Fig. 8:**
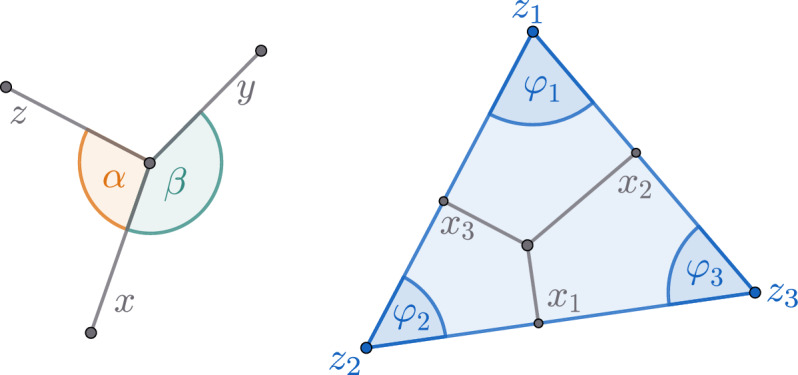
**Left:** Three edges meeting at general angles $\alpha$ and $\beta$ with general weights *x*, *y*, *z* as in the definition of the matrix *M*. **Right:** The special case considered in Lemma 2.3 corresponds to $\alpha =\pi -\varphi _2$ and $\beta =\pi -\varphi _3$, and to weights are as in (A.1). The grey edges meet the blue edges at right angles

**Fig. 9:**
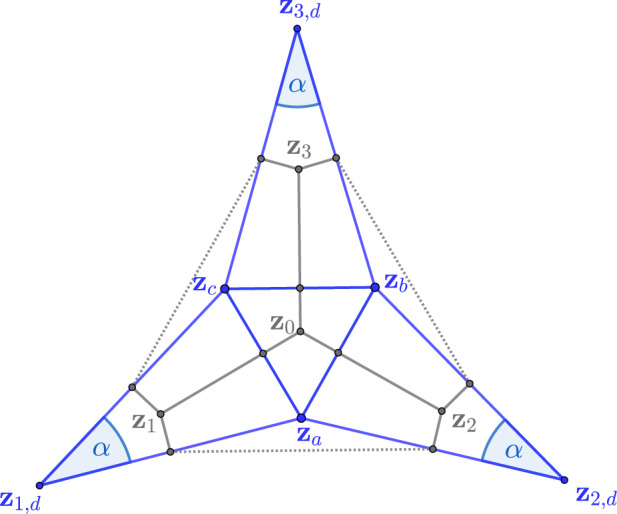
A planar decomposition of a tetrahedron with an equilateral base and isosceles lateral faces with angle $\alpha$ at the apex. The faces are indexed by 0, 1, 2, 3 and the vertices by *a*, *b*, *c*, *d*

## Data Availability

Data sharing is not applicable to this article as no new data were created or analysed in this study.

## Proof of Lemma 2.3

In this section we elaborate on the computation needed to prove Lemma 2.3. To this end, let $x,y,z>0$ and $\alpha ,\beta >0$ such that $\alpha +\beta <2\pi$. We consider the Hermitian matrix

$$\begin{aligned} M=\begin{pmatrix} 0 &  e^{\tfrac{i}{2}(\beta -\pi )} xy &  e^{-\tfrac{i}{2}(\alpha -\pi )}xz \\ e^{-\tfrac{i}{2}(\beta -\pi )} xy & 0 &  e^{-\tfrac{i}{2}(\alpha +\beta -\pi )}yz \\ e^{\tfrac{i}{2}(\alpha -\pi )}xz &  e^{\tfrac{i}{2}(\alpha +\beta -\pi )}yz &  0 \end{pmatrix}. \end{aligned}$$

This matrix is a more general version of the matrix from Lemma 2.3 with arbitrary angles between the three relevant edges and with arbitrary positive weights, see Fig. 8. Let $t=\sqrt{x^2y^2+x^2z^2+y^2z^2}>0$. A computation yields that *M* has eigenvalues $\lambda \in \{ 0,-t,t \}$ with eigenvectors

$$\begin{aligned} \rho (\lambda ) \propto \begin{pmatrix} \lambda ^{2} - y^{2}z^{2} \\ xy\,e^{-\tfrac{i}{2}\beta }\,\big (z^{2}+ i\lambda \big ) \\ xz\,e^{\tfrac{i}{2}\alpha }\,\big (y^{2}- i\lambda \big ), \end{pmatrix} \end{aligned}$$

where $\propto$ denotes equality up to a complex multiplicative constant.

The case $t=1$ is special in that it is equivalent to the existence of three angles $\varphi _1,\varphi _2,\varphi _3>0$ with $\varphi _1+\varphi _2+\varphi _3=\pi$ such that

A.1 $$\begin{aligned} x=x_1:=\sqrt{\tan \frac{\varphi _1}{2}}, \qquad y=x_2:=\sqrt{\tan \frac{\varphi _2}{2}}, \qquad z=x_3:=\sqrt{\tan \frac{\varphi _3}{2}}. \end{aligned}$$

If we moreover assume that $\alpha =\pi -\varphi _2$ and $\beta =\pi -\varphi _3$ as in Fig. 8, we recover the setup of Lemma 2.3. Using standard trigonometric identities we can simplify the formula to get

$$\begin{aligned}&\rho (1) \propto \begin{pmatrix} \sqrt{\sin \varphi _{1}} \\ \sqrt{\sin \varphi _{2}} \\ \sqrt{\sin \varphi _{3}} \end{pmatrix} \propto \begin{pmatrix} |z_{3,2}|^{1/2} \\ |z_{1,3}|^{1/2} \\ |z_{2,1}|^{1/2} \end{pmatrix}, \quad \\  &\rho (-1) \propto \begin{pmatrix} \sqrt{\sin \varphi _{1}} \\ e^{i(\varphi _{3}-\pi )}\,\sqrt{\sin \varphi _{2}} \\ e^{-i(\varphi _{2}-\pi )}\sqrt{\sin \varphi _{3}} \end{pmatrix} \propto \begin{pmatrix} \overline{z_{3,2}}/|z_{3,2}|^{1/2}\\ \overline{z_{1,3}}/|z_{1,3}|^{1/2} \\ \overline{z_{2,1}}/|z_{2,1}|^{1/2} \end{pmatrix}, \end{aligned}$$

where $z_{i,j}=z_i-z_j$, and where the identities follow from the law of sines. The statement of Lemma 2.3 follows readily.

## A Computation of a Loop Holonomy

Here we explicitly compute the holonomy of the connection $\Upsilon$ when the immersion is the symmetric tetrahedron described in Fig. 9, and the loop traverses around the apex. Even though this is a special case of Theorem 1.1, it may be useful for the reader to see a direct computation in such a simple setup.

Up to symmetries, there are two different elementary loops, namely 123 and 012. We focus on 123 and leave the other case for the interested reader. We write $\Upsilon _{123}=\Upsilon _{12}\Upsilon _{23} \Upsilon _{31}$ and our goal is to check that $\Upsilon _{123}=\textrm{Id}$. To this end, let $\vartheta$ be the angle between the outward pointing normals to any two lateral faces of the tetrahedron. By a classical relation between dihedral and face angles in a tetrahedron (see e.g. [35, Eq.(2)]), we have

B.1 $$\begin{aligned} \cos \vartheta =-\frac{\cos \alpha }{1+\cos \alpha }, \quad \text {and therefore} \quad \cos \frac{\vartheta }{2} = \frac{1}{2\cos \frac{\alpha }{2}}, \end{aligned}$$

where the second identity follows from the first one, the half-angle formula for cosine and also the fact that $\vartheta \in (-\pi ,\pi ]$ (which fixes the sign).

From (2.8) we have

$$\begin{aligned} \Upsilon ^{-1}_z(\psi _{12})\Upsilon _{123}\Upsilon _z(\psi _{12})&= [\Upsilon _x(\vartheta )\Upsilon _z(\phi _{12})\Upsilon _z(\psi _{23})]\\  &\qquad [\Upsilon _x(\vartheta )\Upsilon _z(\phi _{23})\Upsilon _z(\psi _{31})] [\Upsilon _x(\vartheta )\Upsilon _z(\phi _{31})\Upsilon _z(\psi _{12})] \\&= [\Upsilon _x(\vartheta )\Upsilon _z(\phi _{12}+\psi _{23})] [\Upsilon _x(\vartheta )\Upsilon _z(\phi _{23}+\psi _{31})]\\  &\qquad [\Upsilon _x(\vartheta )\Upsilon _z(\phi _{31}+\psi _{12})] \\&= [\Upsilon _x(\vartheta )\Upsilon _z(2\pi -\alpha )]^3, \end{aligned}$$

where the third identity follows from the fact that

$$ \phi _{12}+\psi _{23}= \pi -\beta _{21}+\beta _{23}= \pi -\arg (\textbf{z}_{2,d}-\textbf{z}_{a})+\arg (\textbf{z}_{b}-\textbf{z}_{2,d})=2\pi -\alpha , $$

which in turn is a consequence of (2.7) and elementary geometry. By the rotational symmetry of the picture, the same is true for the other two pairs of faces.

It is therefore enough to show that $M^3:= [\Upsilon _x(\vartheta )\Upsilon _z(2\pi -\alpha )]^3=\textrm{Id}$. To this end, we note that since every $\text {SU}(2)$ matrix *M* is conjugate to a diagonal matrix $$ \begin{pmatrix} e^{i\gamma } &  0 \\ 0 &  e^{-i\gamma } \end{pmatrix}$$, the equation $M^3=\textrm{Id}$ is equivalent to both $e^{i\gamma }$ and $e^{-i\gamma }$ being cube roots of unity, and hence we must have $\gamma =\pm \frac{2\pi }{3}$ or $\gamma =0$. The case $\gamma =0$ can be excluded in our situation, and therefore $M^3=\textrm{Id}$ simplifies to ${{\,\textrm{Tr}\,}}M=2\cos \frac{2\pi }{3}= -1$. On the other hand we notice that

$$ {{\,\textrm{Tr}\,}}[\Upsilon _x(\vartheta )\Upsilon _z(2\pi -\alpha )]=2\cos \frac{\vartheta }{2} \cos \frac{2\pi -\alpha }{2}=-2\cos \frac{\vartheta }{2} \cos \frac{\alpha }{2}=-1, $$

where the last identity follows from (B.1). Altogether this means that $\Upsilon _{123}=\textrm{Id}$ which is in accordance with Theorem 1.1.
